# Long-term correction of hemophilia A via integration of a functionally enhanced *FVIII* gene into the *AAVS1* locus by nickase in patient-derived iPSCs

**DOI:** 10.1038/s12276-024-01375-z

**Published:** 2025-01-06

**Authors:** Do-Hun Kim, Sang-Hwi Choi, Jin Jea Sung, Sieun Kim, Hanui Yi, Sanghyun Park, Chan Wook Park, Young Woo Oh, Jungil Lee, Dae-Sung Kim, Jong-Hoon Kim, Chul-Yong Park, Dong-Wook Kim

**Affiliations:** 1 Department of Physiology, Yonsei University College of Medicine, 50-1 Yonsei-ro, Seodaemun-gu, Seoul, 03722 Korea; 2 S. Biomedics Co., Ltd, 28 Seongsui-ro 26-gil, Seongdong-gu, Seoul, 04797 Korea; 3 Brain Korea 21 PLUS Program for Medical Science, Yonsei University College of Medicine, 50-1 Yonsei-ro, Seodaemun-gu, Seoul, 03722 Korea; 4 Department of Biotechnology, College of Life Sciences and Biotechnology, Korea University, 145 Anam-ro, Seongbuk-gu, Seoul, 02841 Korea

**Keywords:** Induced pluripotent stem cells, Genetic engineering, Targeted gene repair, Stem-cell research, Haematological diseases

## Abstract

Hemophilia A (HA) is caused by mutations in coagulation *factor VIII* (*FVIII*). Genome editing in conjunction with patient-derived induced pluripotent stem cells (iPSCs) is a promising cell therapy strategy, as it replaces dysfunctional proteins resulting from genetic mutations with normal proteins. However, the low expression level and short half-life of FVIII still remain significant limiting factors in the efficacy of these approaches in HA. Here, we constructed a functionally enhanced *FVIII* variant, F309S/E1984V-mutated B domain-deleted (BDD)-*FVIII* (*FE-FVIII*), with increased activity and stability. We inserted *FE-FVIII* with a human elongation factor-1 alpha (EF1α) promoter into the *AAVS1* locus of HA patient-derived iPSCs via CRISPR/Cas9 (D10A) nickase to ensure expression in any cell type. *FE-FVIII* was expressed not only in undifferentiated *FE-FVIII*-inserted (FE-KI) iPSCs but also in endothelial cells (ECs) differentiated from them in vitro. Compared with mice transplanted with wild-type *BDD-FVIII*-containing ECs, immunocompetent HA mice intravenously transplanted with FE-KI ECs presented a 2.12-fold increase in FVIII activity in the blood and an approximately 20% greater survival rate after hemorrhagic tail injury. For sustained efficacy, FE-KI ECs were subcutaneously transplanted into immunodeficient HA mice, resulting in amelioration of the hemophilia phenotype for more than 3 months. This strategy can improve FVIII function and may provide a universal therapeutic approach for treating HA.

## Improved FVIII expression offers hope for hemophilia A

Hemophilia A is a genetic disorder that causes bleeding due to a faulty gene on the X chromosome. Researchers　explored a new approach using stem cells and gene editing to improve treatment. They used induced pluripotent stem cells, which can become any cell type, from HA patients. By editing these cells with CRISPR/Cas9, they inserted a modified version of the faulty gene into a safe spot in the DNA. This study involved lab experiments and tests on mice to see if the edited cells could produce the necessary protein, Factor VIII, more effectively. The modified FVIII showed better activity and stability than the regular version. Mice treated with these cells had improved blood clotting and survival rates after injury. The researchers concluded that this method could be a promising step toward better treatments for HA.

This summary was initially drafted using artificial intelligence, then revised and fact-checked by the author.

## Introduction

Hemophilia A (HA) is a relatively common genetic bleeding disorder caused by various mutations in the coagulation *factor VIII* (*FVIII*) gene on chromosome X^1^. In the coagulation pathway, active FVIII binds with active FIX to form a tenase complex, which activates FX. As the tenase complex initiates a positive feedback loop of coagulation, FVIII is an essential protein in this pathway^2^. To date, there is no fundamental cure for HA. Patients with severe HA (<1% FVIII activity) are prone to chronic musculoskeletal disorders and internal bleeding^3^ and require expensive, life-long treatment with recombinant FVIII every 2 or 3 days^4^.

Patient-derived induced pluripotent stem cells (iPSCs) offer a promising avenue for developing new treatments for HA. iPSCs can be generated directly from somatic cells via the induction of reprogramming factors such as *OCT4, SOX2, KLF4*, and *c-MYC*^5^. Since iPSCs can be differentiated into any type of cell, they serve as an excellent source for cell therapies aimed at replacing cells or tissues that do not function normally. Recently, it was confirmed that FVIII could be successfully expressed in endothelial cells (ECs) differentiated from patient-derived iPSCs in which an *FVIII* gene inversion was corrected using programmable nucleases^6^. However, more than half of patients with HA have other genetic mutations in *FVIII*, including large deletions, insertions, duplications, or point mutations^7^. Therefore, there is a need for universal gene therapy that can address all types of genetic mutations that occur in HA^8,9^.

Genome editing with programmable nucleases enables straightforward modification of targeted DNA. Programmable nucleases, including zinc finger nucleases (ZFNs), transcription activator-like effector nucleases (TALENs), and clusters of regularly interspaced palindromic repeats (CRISPR)/Cas9, can produce site-specific double-stranded breaks (DSBs) in DNA. These DSBs are repaired by homology-directed repair (HDR) or nonhomologous end joining (NHEJ), which are endogenous repair mechanisms that can lead to genetic alterations such as insertions, deletions, and chromosomal rearrangements and can be used for gene editing^10,11^. The combination of iPSCs and genome editing with programmed nucleases has tremendous potential for new cell therapies to treat genetic diseases^12^.

Because FVIII has a short half-life of 12 h^13^, various strategies have been investigated to improve treatment options for patients with HA^14^. The development of FVIII with an enhanced half-life to prolong the duration at which FVIII remains in circulation is one of the oldest strategies and is still ongoing to improve HA treatment. Although cell therapies using ECs differentiated from pluripotent stem cells (PSCs) constitute another potential strategy for treating HA, their limited therapeutic effects, which are caused mainly by the low endogenous expression and activity of secreted FVIII, remain challenging. To overcome these limitations, we have developed functionally enhanced FVIII to increase both the activity and half-life of FVIII.

In this study, we employed CRISPR/Cas9 (D10A) nickase-mediated knock-in to insert either the wild-type (WT) B domain-deleted (BDD)-*FVIII* gene or a functionally enhanced *BDD*-*FVIII* gene (F309S/E1984V-mutated *BDD-FVIII*, *FE-FVIII*) driven by a human elongation factor-1 alpha (EF1α) promoter into the adeno-associated virus site 1 (*AAVS1)* locus of iPSCs derived from a patient with HA. This insertion resulted in constant expression of *FVIII* mRNA and increased FVIII activity in both the iPSCs and the ECs differentiated from the genetically modified iPSCs. Additionally, secreted FE-FVIII demonstrated greater activity and a longer half-life than did secreted WT BDD-FVIII. HA mice transplanted with ECs containing *FE-FVIII* presented greater FVIII activity in their blood than those transplanted with ECs carrying WT *BDD-FVIII*. Furthermore, compared with HA mice or mice transplanted with ECs without gene correction, mice transplanted with ECs corrected with *FE-FVIII* exhibited improved survival rates after hemorrhagic tail injury. Long-term efficacy studies in immunodeficient hemophilic mice further corroborated the elevated FVIII activity.

## Materials and methods

### Cell cultures

Human embryonic kidney (HEK) 293 T cells (ATCC, Manassas, VA, USA) were maintained in Dulbecco’s modified Eagle’s medium (DMEM; Gibco, Grand Island, NY, USA) supplemented with 10% (vol/vol) fetal bovine serum (FBS; HyClone, Logan, UT, USA) and 1% (vol/vol) penicillin-streptomycin (Gibco). Intron 22-inverted patient-derived iPSCs^6^ and iPSCs with WT *BDD-FVIII* insertion or *FE-FVIII* insertion were maintained on Matrigel (Corning, Corning, NY, USA)-coated culture dishes in StemMACS^TM^ iPSC-Brew XF medium (StemMACS) (Miltenyi Biotec, Bergisch Gladbach, Germany) according to the manufacturer’s instructions for feeder-free culture.

### Preparation of donor plasmids and guide RNA for SpCas9 nickase

To construct donor plasmids, the pcDNA4/BDD-FVIII plasmid (Addgene, www.addgene.org, #41035) was used as a backbone^15^. The 5’-homology arm (left arm, LA) and 3’-homology arm (right arm, RA) were designed in a previous study^16^ and inserted into the MunI/MluI and PacI/MauBI sites, respectively. The human EF1α promoter sequence was cloned between the left arm and the *FVIII* open reading frame using the MluI/RruI site. A bovine growth hormone (bGH) polyadenylation signal and puromycin resistance cassette flanked by *loxP* sites were inserted between the *FVIII* gene cassette and the right arm by the Gibson Assembly^®^ Cloning Kit (New England Biolabs, Massachusetts, UK). F309S, E1984V, and F309S/E1984V variants were constructed by Gibson assembly. The sequences of the cloned DNA were confirmed by Sanger sequencing at Cosmogenetech (Seoul, Korea). *Streptococcus pyogenes* Cas9 (SpCas9) (D10A) nickase and 5′-GX19 sgRNA (5′-GGGCCACTAGGGACAGGAT-3′) expression plasmids were purchased from ToolGen (Seoul, Korea).

### RNA isolation, quantitative real-time PCR (qPCR), and reverse transcription PCR (RT‒PCR) analysis

Total RNA was isolated from cells using the Easy-Spin^TM^ Total RNA Extraction Kit (iNtRON Biotechnology, Seongnam, Korea) according to the manufacturer’s instructions. cDNA was synthesized from 1 μg of total RNA using the PrimeScript^TM^ RT Master Mix (Takara Bio, Kusatsu, Japan). For quantification of the mRNA levels, qPCR was performed using SYBR^®^ Premix Ex-Taq (Takara Bio) and a CFX96 Real-Time System (Bio-Rad, Hercules, CA, USA). Ct values for each gene were normalized to the Ct values of *GAPDH*. RT‒PCR was performed using the EmeraldAmp^®^ GT PCR Master Mix (Takara Bio). The primers used for RT‒PCR and qPCR are listed in Supplementary Table 1.

### Generation of *FVIII* gene-inserted iPSCs

The iPSCs derived from a patient with HA were washed once with DPBS and dissociated into single cells using ReLeSR^TM^ (STEMCELL Technologies, Vancouver, Canada). Then, 5 × 10^5^ iPSCs were electroporated with 1 μg of Cas9 nickase, 2 μg of sgRNA expression vector, and 2 μg of each donor plasmid via a Neon transfection system (Invitrogen, Carlsbad, CA, USA). After being pulsed once at 1000 V for 30 ms, the cells were cultured in StemMACS medium supplemented with 10 μM Y-27632 (Sigma‒Aldrich, St. Louis, MO, USA) for 2 days. Four days after transfection, the cells were selected with 0.5 μg/mL puromycin. To isolate clonal populations of genetically modified iPSCs, three rounds of single-colony expansion with puromycin were performed. After isolation of the genetically modified iPSCs, 5 × 10^5^ cells were electroporated with 1 μg pCAG-Cre:GFP vector (Addgene, #13776) to remove the puromycin resistance cassette, and clonal selection was performed for each iPSC colony 10 days after electroporation. To trace the long-term survival of transplanted cells, we generated a luciferase KI reporter line by replacing BDD-FVIII in the donor plasmid with luciferase and performing a knock-in into the same patient-derived iPSCs as described above. This study was approved by the Yonsei University Institutional Review Board (IRB #4-2012-0028).

### PCR analysis and DNA sequencing of knock-in junctions in genetically modified iPSCs

Genomic DNA was isolated from cells using the DNeasy Blood & Tissue Kit (QIAGEN, Hilden, Germany) according to the manufacturer’s instructions. To identify the knock-in of donor DNA into the *AAVS1* locus, DNA fragments for each junction were amplified using the EmeraldAmp^®^ GT PCR Master Mix. The sequence of each DNA amplicon was verified by Sanger sequencing at Cosmogenetech. The primers used for genotyping, and Sanger sequencing is listed in Supplementary Table 1.

### In vitro differentiation into the three germ layers

An in vitro three-germ layer-formation assay was performed as previously described^17^. Briefly, iPSC colonies were dissected and detached to generate embryoid bodies (EBs) and then cultured in low-attachment cell culture dishes in EB culture medium [DMEM/F12 medium (Gibco) with 4 ng/mL basic fibroblast growth factor (bFGF; PeproTech, Rocky Hill, JN, USA), 20% knockout serum replacement (Invitrogen), 1% nonessential amino acids (Invitrogen), and 0.1 mM 2-mercaptoethanol (Sigma‒Aldrich)] supplemented with 5% FBS for 1 week. EBs were then plated onto Matrigel-coated dishes and cultured for an additional 2 weeks for spontaneous differentiation.

### Targeted deep sequencing for off-target analysis

Four potential off-target sites differing by up to three nucleotides from the on-target site were identified using a web-based in silico tool (www.rgenome.net)^18^. For targeted deep sequencing, genomic DNA was isolated from patient-derived parental iPSCs and genetically modified iPSC lines using DNeasy Blood & Tissue Kits (QIAGEN), and off-target regions were amplified and analyzed using a MiSeq system (Illumina, San Diego, CA, USA) at ToolGen. The primers used for off-target amplification are listed in Supplementary Table 2.

### Differentiation into ECs

To induce the differentiation of iPSCs into ECs, we used a previously described protocol with minor modifications^19^. Briefly, iPSCs were dissociated with ReleSR^TM^ and cultured in Matrigel-coated culture dishes in StemMACS medium supplemented with 10 μM Y-27632 for 1 day. The culture medium was then changed to STEMdiff^TM^ APEL^TM^2 medium (STEMCELL technologies) with 6 μM CHIR99021 (Tocris Bioscience, Bristol, UK), and the cells were incubated for another 2 days to induce the mesoderm lineage. On Day 2, the cells were cultured in STEMdiff^TM^ APEL^TM^ 2 medium supplemented with 25 ng/mL bone morphogenetic protein 4 (BMP4; Prospec, East Brunswick, NJ, USA), 10 ng/mL bFGF, and 50 ng/mL vascular endothelial growth factor (VEGF)-A (PeproTech) for 2 days to induce the vascular lineage. On Day 4, the cells were detached with TrypLE^TM^ Select Enzyme (Gibco), transferred to new culture dishes, and cultured in EC growth medium-MV2 (ECGM-MV2; Promocell, Heidelberg, Germany) supplemented with 50 ng/mL VEGF-A for another 4 days to generate endothelial progenitors, with the media changed every other day.

### Immunocytochemistry and karyotyping

For immunofluorescence staining, the cells were fixed in a 4% paraformaldehyde solution for 15 min at room temperature (RT), washed three times with DPBS, and permeabilized with 0.1% Triton X-100 in PBS for 10 min at RT. The cells were then washed three times with PBS and incubated in a blocking buffer (2% bovine serum albumin in PBS) for 1 h at RT. The cells were then incubated with primary antibodies in a blocking buffer for 1 h at RT. The following antibodies were used: mouse anti-SSEA-4 (1:200, Millipore, Billerica, MA, USA), rabbit anti-OCT4 (1:200, Santa Cruz Biotechnology, Dallas, TX, USA), rabbit anti-NESTIN (1:1000, Millipore), goat anti-HNF3β (1:200, Santa Cruz Biotechnology), mouse anti-α-SMA (1:400, Sigma‒Aldrich), mouse anti-CD31 (1:200, BD Biosciences, San Jose, CA, USA), and rabbit anti-VWF (1:500, Millipore). The cells were then washed three times with PBS and incubated with fluorescence-tagged secondary antibodies (Alexa Fluor^®^ 488 or 594, 1:1000, Invitrogen) in a blocking buffer for 30 min at RT. The cells were then washed three times with PBS and mounted onto coverslips using 4′,6-diamidino-2-phenylindole (DAPI) mounting medium (Vector Laboratories, Burlingame, CA, USA). Images were captured with a fluorescence microscope (Eclipse Ti-U, Nikon Instruments Inc., Tokyo, Japan). For karyotyping, the chromosomes from each iPSC line were stained with Giemsa for G-banding analysis and analyzed at EONE laboratories (Incheon, Korea). The list of antibodies used is summarized in Supplementary Table 3.

### Measurement of FVIII activity and total FVIII antigen

To measure FVIII activity, 293 T cells and iPSCs were cultured in phenol red-free DMEM/F12 medium (Gibco) for 24 h at 37 °C, and ECs were cultured in phenol red-free ECGM-MV2 medium supplemented with 50 ng/mL VEGF-A for 48 h at 37 °C. Supernatants from the iPSCs and ECs were collected and concentrated 30-fold using an Amicon^®^ Ultra15 centrifugal filter (Millipore). FVIII activity in the culture supernatants was measured using a Coamatic^®^ Factor VIII chromogenic assay kit (Instrumentation Laboratory, Bedford, MA, USA) according to the manufacturer’s instructions. FVIII activity measurements were performed using a 96-well microplate, and the absorbance at 405 nm was measured using a microplate reader (Molecular Devices, San Jose, CA, USA). A standard curve was generated with HemosIL^®^ Calibration Plasma (Instrumentation Laboratory). To determine the secretion of FVIII, ELISA was performed. After the medium was harvested, the cells were collected and lysed with 200 µl of radioimmunoprecipitation assay (RIPA) buffer. The concentrated medium and cell lysates were then analyzed using a human FVIII ELISA kit (total FVIII antigen) (Abcam Cambridge, UK) following the manufacturer’s instructions.

### In vivo cell transplantation into HA mice

HA mice (strain B6;129S4-F8^*tm1Kaz*^/J) were purchased from Jackson Laboratory (Bar Harbor, ME, USA). For intravenous injection, three-month-old HA mice were injected with 1 × 10^6^ parental or genetically modified ECs via the tail vein. The mice were treated with cyclosporine A (210 mg/L, in drinking water) starting 3 days before transplantation. Blood samples were collected from the tail vein 3, 5, 7, 10, and 14 days after transplantation for measurements of FVIII expression and activity. F8-deficient immunodeficient mice (NOD-Prkd^cem1Gmcr^ Il2rg^em1Gmcr^ F8^em1Gmcr^, N2G-F8^em1Gmcr^) were purchased from Gemcro (Seoul, Korea). To confirm the long-term survival of subcutaneously transplanted cells, 4 × 10^6^ ECs derived from luciferase-KI reporter iPSCs were subcutaneously transplanted into the thighs of immunodeficient mice. Imaging was conducted weekly from the day after transplantation via an IVIS 200 luminescence and fluorescence animal imaging system (Perkin Elmer, Shelton, CT, US). Twenty minutes before imaging, IVISbrite D-Luciferin (PerkinElmer, Connecticut, USA) was injected intraperitoneally. To verify long-term efficacy, eight-week-old N2G-F8^em1Gmcr^ mice received subcutaneous transplantation of 4 × 10^6^ parental or genetically corrected ECs, and blood samples were collected at 4 and 13 weeks posttransplantation to measure FVIII activity in the plasma. All surgeries and experiments were performed with the approval of the Institutional Animal Care and Use Committee of Yonsei University College of Medicine, Seoul, Korea (2012-0215).

### Tail clip challenge

A tail clip challenge was performed on anesthetized mice as described in a previous report^6^ with minor modifications. In brief, the distal part of the tail (2 mm diameter) was cut and allowed to bleed for 5 min. Firm pressure was then applied to the tail for 1 min. The survival time was subsequently monitored until 2 days after clipping, which was the experimental endpoint.

### Decay rate and half-life analysis

The FVIII activity as a function of time was fitted to a single exponential decay curve via nonlinear least squares regression via the equation *A* = *A*_0_ × *e*^−*kt*^, where *A* is the residual FVIII activity, *A*_0_ is the initial FVIII activity, *k* is the apparent rate constant, and *t* is the length of incubation (in hours) at 37 °C. The half-life of FVIII was calculated as the time when *A* = 1/2*A*_0_ and expressed as a relative value.

### Statistical analysis

All the data are expressed as the means ± SEMs of at least three independent experiments. Statistical analysis was performed via GraphPad Prism software (GraphPad Software, San Diego, CA, USA). Multiple comparisons were analyzed by one-way ANOVA with a post hoc test. The statistical significance of differences between the two groups was determined by Student’s *t* test. Survival curves were compared via the log-rank test.

## Results

### Construction and selection of donor plasmids

To overcome the low therapeutic efficacy of EC-secreted FVIII, we designed four donor plasmids expressing different variants of *FVIII* under the control of the human elongation factor-1 alpha (EF1α) promoter, with a puromycin resistance cassette in the plasmid backbone. Previous studies have reported that bioengineering FVIII improves its properties^20^. Among these, we selected the F309S (F) and E1984V (E) variants, which have been shown to increase the secretion^21^ and stability^22^ of FVIII, respectively. We also generated the F309S/E1984V (FE) variant by combining the F and E variants. A nonmutated WT *BDD-FVIII* gene and *FVIII* genes containing F, E, and FE variants were inserted into the respective plasmids. We then assessed the activity of each FVIII variant via an FVIII activity assay (Supplementary Fig. 1a). We transfected 293 T cells with each FVIII variant, incubated the cells at 37 °C for 24 h, and harvested and analyzed the culture supernatants. Compared with those of the WT BDD-FVIII, the activity levels of the supernatants containing F and FE increased 1.17-fold and 1.20-fold, respectively, whereas the activity level of the supernatant of E decreased 0.75-fold.

We then estimated the stability of the FVIII variants by a decay assay in which FVIII activity was measured in supernatants from each cell line after additional cell-free incubation at 37 °C for 0–24 h (Supplementary Fig. 1b). The results showed that the stability of the F variant was not significantly different from that of WT BDD-FVIII, whereas the E and FE variants both had increased half-lives (Supplementary Fig. 1c). Thus, these results demonstrated that the FE variant increased both the activity and stability of FVIII.

### Targeted knock-in of WT *BDD*-*FVIII* and *FE-FVIII* into the *AAVS1* locus of iPSCs derived from patients with HA

**Fig. 1:**
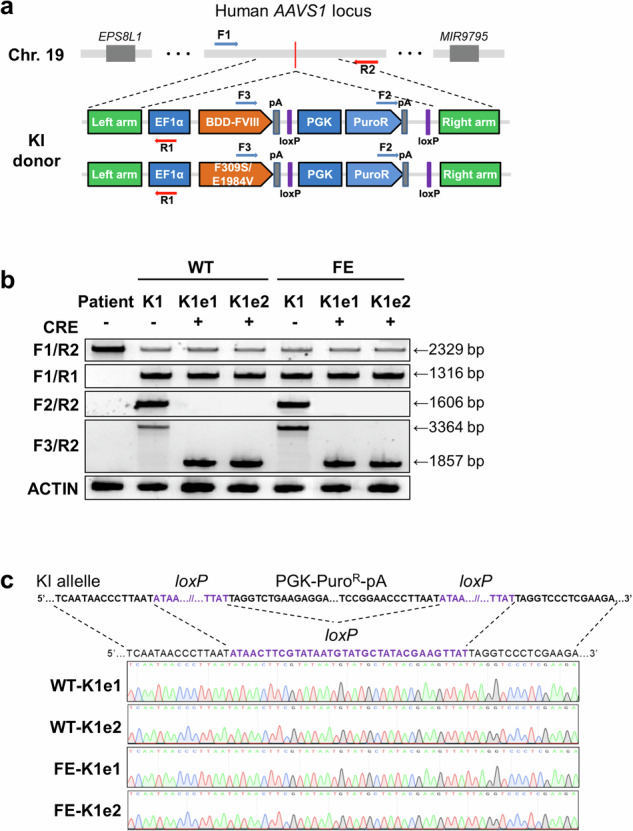
Targeted insertion of *FVIII* genes into the *AAVS1* locus in iPSCs derived from a patient with HA. **a** Schematic view of target sites for primary PCR screening after knock-in into the *AAVS1* locus. The primers used in the PCR-based genotype analysis are represented by arrows. **b** PCR-based genotype analysis to identify the removal of the puromycin resistance cassette from genetically modified iPSC lines. The F1/R1 and F2/R2 primer sets were used to detect the 5′ junction and 3′ junction, respectively. The F3/R2 primer set was used to detect the excision of the puromycin resistance cassette. **c** DNA amplicons generated with the F3/R2 primer set showing the sequences around the *loxP* site in the genetically modified iPSC lines after excision of the resistance cassette.

Next, we generated iPSCs containing each FVIII variant. To insert WT *BDD-FVIII* or *FE-FVIII* into the *AAVS1* locus, we used a previously reported single guide RNA (sgRNA) targeting intron 1 of the *PPP1R12C* gene^16^ (Supplementary Fig. 2a). Each donor plasmid, together with the sgRNA and SpCas9 nickase (D10A) vectors, was electroporated into iPSCs derived from a patient with HA (Fig. 1b). After puromycin selection and additional culture, genomic DNA was extracted from drug-resistant iPSC colonies, and knock-in clones were confirmed via genotyping PCR analysis as previously described^23^. To screen for successful knock-in clones, we amplified the 5’ junction and 3’ junction of the knock-in site with specific primer sets (F1/R1, F2/R2; Fig. 1a). Four of the 88 puromycin-resistant clones with the WT *BDD-FVIII* donor plasmid (4.54%) and five of the 92 puromycin-resistant clones with the *FE-FVIII* donor plasmid (5.43%) presented amplified PCR bands for both the 5′ and 3′ junctions. We subjected these clones to two rounds of single-colony expansion and selected two clones containing each FVIII variant (WT-K1, WT-K2, FE-K1, and FE-K2; Supplementary Fig. 2b). We further verified the targeted insertions of each *FVIII* gene by Sanger sequencing of the PCR products (Supplementary Fig. 2c).

We then excised the puromycin resistance cassette from the WT-K1 and FE-K1 iPSC lines. After transient *Cre* recombinase expression was induced via electroporation, we screened clones from each iPSC line via PCR products amplified with the F3/R2 primers (Fig. 1a). The results revealed that the puromycin resistance cassette was excised from three out of eight WT-K1 clones (37.5%) and four out of eight FE-K1 clones (50%). We selected two iPSC lines derived from WT-K1 (WT-K1e1 and WT-K1e2) and two iPSC lines derived from FE-K1 (FE-K1e1 and FE-K1e2), for which Sanger sequencing confirmed the complete removal of the puromycin resistance cassette (Fig. 1b, c).

### Pluripotency and off-target analysis of *FVIII* knock-in iPSC lines

**Fig. 2:**
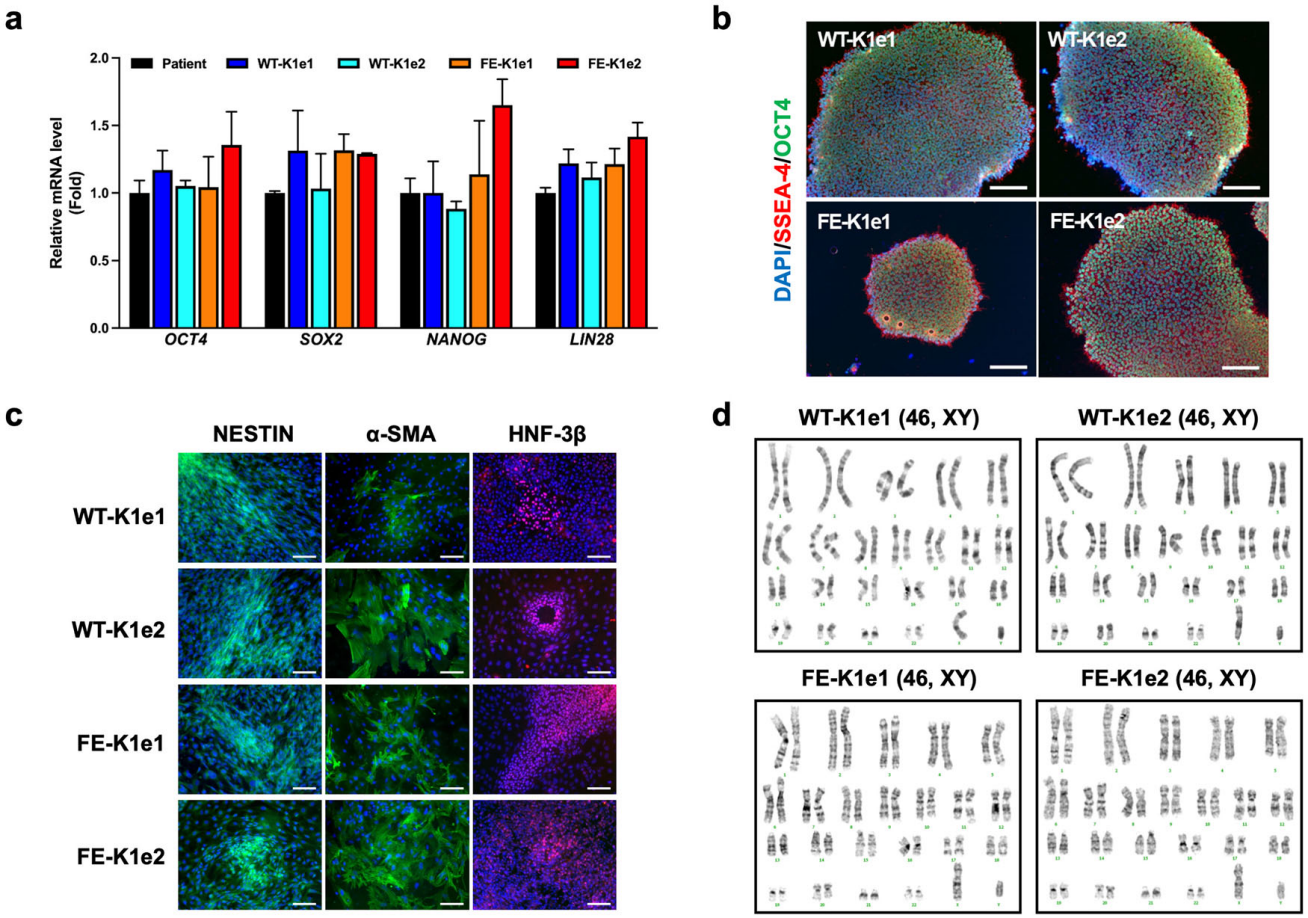
Pluripotency marker expression in knock-in iPSC lines. **a** Quantitative real-time PCR (qPCR) analysis of pluripotency markers (*OCT4, SOX2, NANOG*, and *LIN28*) in parental and genetically modified iPSC lines. *GAPDH* was used to normalize gene expression. The data are presented as the means ± SEMs of three independent experiments. **b** Immunofluorescence staining showing the expression of pluripotency marker proteins (OCT4 and SSEA-4) in genetically modified iPSC lines. Nuclei were labeled with DAPI (scale bars, 100 μm). **c** Immunofluorescence staining showing the expression of marker proteins for the ectoderm (NESTIN), mesoderm (α-smooth muscle actin, α-SMA), and endoderm (hepatocyte nuclear factor-3β, HNF-3β) in genetically modified iPSC lines. Nuclei were labeled with DAPI (scale bars, 100 μm). **d** Karyotype analysis was performed in genetically modified iPSC lines.

Next, we investigated whether the *FVIII* knock-in iPSC lines maintained their pluripotency compared with their parental iPSCs. The results of quantitative real-time PCR (qPCR) revealed that the mRNA expression levels of pluripotency marker genes (*OCT4*, *SOX2*, *NANOG*, and *LIN28*) in the knock-in iPSC lines were similar to those in the parental iPSCs (Fig. 2a). We also detected similar expression levels of pluripotency marker proteins (SSEA-4 and OCT4) in the knock-in and parental iPSC lines via immunostaining (Fig. 2b). The knock-in lines successfully differentiated into each of the three germ layers in vitro, as shown by the expression of ectoderm (NESTIN), mesoderm (alpha-smooth muscle actin, α-SMA), and endoderm (hepatocyte nuclear factor-3beta, HNF-3β) markers (Fig. 2c). In addition, G-banding revealed that all the knock-in iPSC lines had a normal 46, XY karyotype (Fig. 2d).

We then determined whether the Cas9 nickase had introduced any off-target mutations in the knock-in iPSC lines. We used a web-based tool to identify potential off-target sites that differed from the on-target site by up to three nucleotides. We selected four potential off-target sites for targeted deep sequencing in the four knock-in iPSC lines and the parental iPSCs. The results revealed no significant mutations at the off-target sites in the knock-in iPSC lines (Supplementary Fig. 3).

### Restoration of FVIII expression in the knock-in iPSC lines

**Fig. 3:**
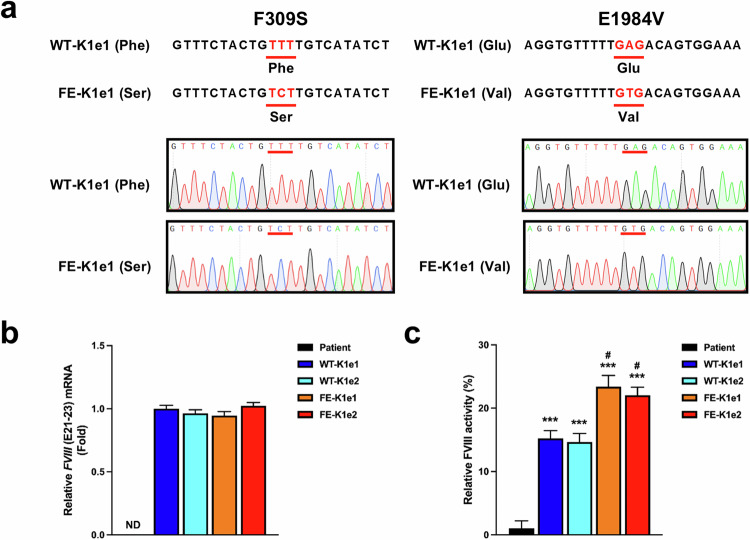
Phenotypic rescue of *FVIII* expression in knock-in iPSC lines. **a** Sanger sequencing of *FVIII* mRNA in genetically modified iPSC lines was performed to determine whether the mutated genetic sequences were transcribed. The 309 Phe and 1984 Glu residues were successfully mutated to 309 Ser and 1984 Val in the FE-KI iPSCs. **b** Results of the qPCR analysis showing the *FVIII* (E21–23) expression levels in the parental and genetically modified iPSC lines. *GAPDH* was used to normalize gene expression. The data are presented as the means ± SEMs of three independent experiments. ND indicates “not detected”. **c** FVIII activity was measured after 30-fold concentration in supernatants harvested from parental or genetically modified iPSC lines. The data represent the activity detected per 1 × 10^5^ iPSCs. The data are presented as the means ± SEMs of three independent experiments. ***p < 0.001 compared with parental iPSCs (one-way ANOVA with post hoc Dunnett’s test) and ^#^*p* < 0.05 compared with WT-KI iPSC lines (one-way ANOVA with post hoc Tukey’s test).

After the successful insertion of WT *BDD-FVIII* or *FE-FVIII* into the *AAVS1* locus, we extracted mRNA from each knock-in line to identify the transcripts of the *FVIII* gene. Sanger sequencing revealed that FE-KI resulted in changes in mRNAs containing the 309 Phe-to-Ser and 1984 Glu-to-Val changes, whereas WT-KI resulted in mRNAs containing the wild-type codons (Fig. 3a).

Next, we confirmed the phenotypic restoration of *FVIII* gene expression in the knock-in iPSC lines in vitro. qPCR analysis revealed high expression of *FVIII* mRNA in the knock-in iPSC lines but not in the parental iPSCs (Fig. 3b). There were no significant differences in the expression levels of *FVIII* mRNA between WT-KI and FE-KI iPSCs; however, activity assays revealed that FVIII activity was approximately 1.5-fold greater in the FE-KI lines (23.38 ± 0.78% in FE-K1e1, 22.04 ± 0.26% in FE-K1e2) than in the WT-KI lines (15.22 ± 0.22% in WT-K1e1, 14.66 ± 0.45% in WT-K1e2) (Fig. 3c).

### Restoration of FVIII expression in knock-in iPSC-derived ECs

**Fig. 4:**
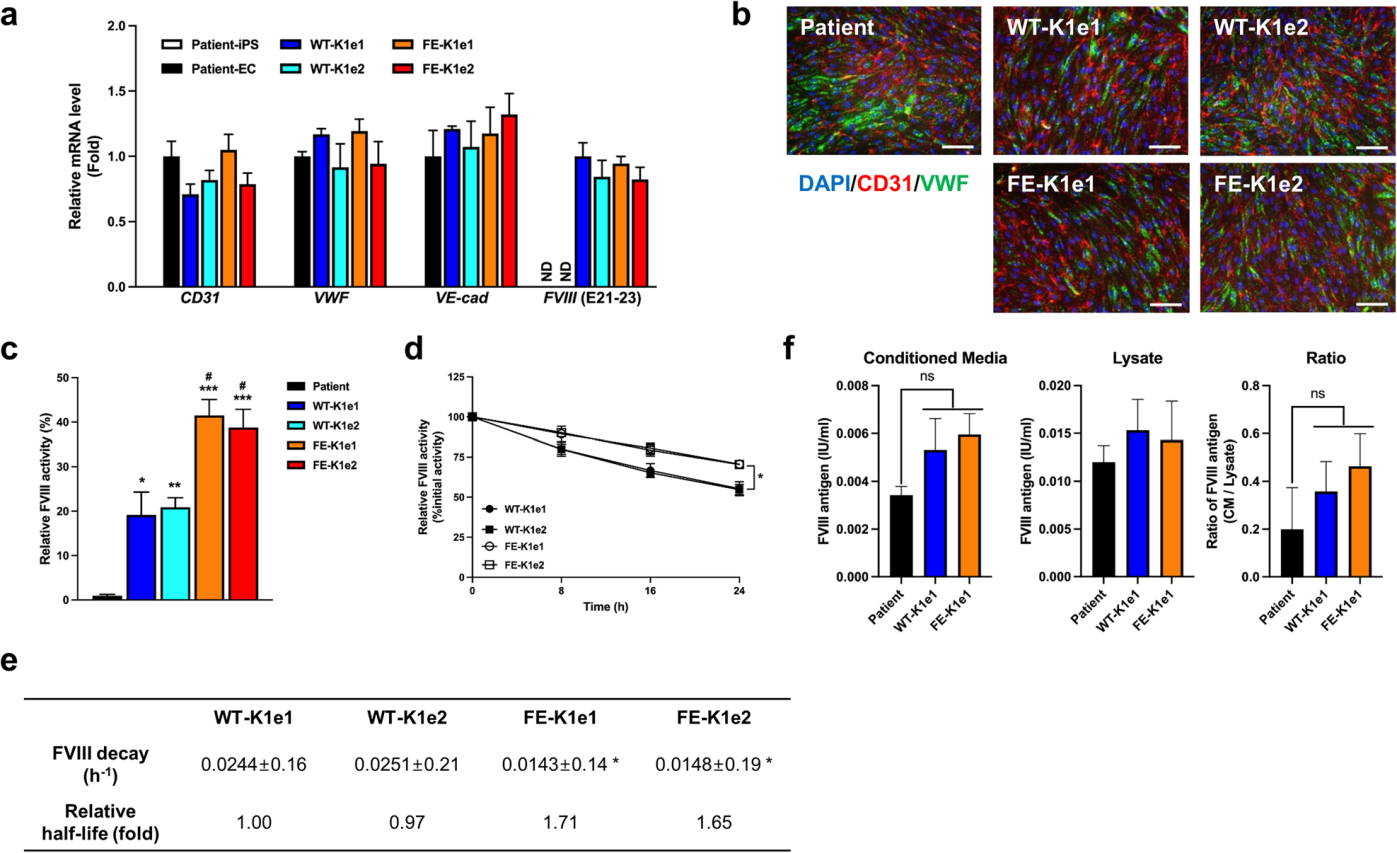
Functional recovery from FVIII deficiency in ECs differentiated from knock-in iPSC lines. **a** Results of qPCR analysis showing the expression levels of EC markers (*CD31*, *VWF*, and *VE-cadherin*) and *FVIII* in ECs differentiated from parental and genetically modified iPSC lines. *GAPDH* was used to normalize gene expression. The data are presented as the means ± SEMs of three independent experiments. ND indicates “not detected”. **b** Immunofluorescence staining revealed the expression of endothelial marker proteins (CD31 and VWF) in ECs differentiated from genetically modified iPSC lines. Nuclei were labeled with DAPI (scale bars, 100 μm). **c** FVIII activity was measured after a 30-fold concentration of supernatants harvested from ECs differentiated from parental or genetically modified iPSC lines. The data represent the activity detected per 1 × 10^5^ ECs. The data are presented as the means ± SEMs of three independent experiments. * *p* < 0.05, ** *p* < 0.01 and *** *p* < 0.001 compared with ECs differentiated from parental iPSCs (one-way ANOVA with post hoc Dunnett’s test) and ^#^*p* < 0.05 compared with WT-KI ECs (one-way ANOVA with post hoc Tukey’s test). **d** Activity in the decay assay was determined after incubating EC-derived FVIII for 0 h, 8 h, 16 h, or 24 h at 37 °C. The data are presented as the means ± SEMs of three independent experiments. *p < 0.05 compared with WT-KI ECs (two-way ANOVA with post hoc Tukey’s test). **e** FVIII decay rate and relative half-life of FVIII harvested from ECs. The data are the means ± SEMs of three independent experiments. SDs for rate decay values are estimated via least squares curve fitting and are within approximately 10% of the mean values. *p < 0.01 compared with WT-KI ECs (one-way ANOVA with post hoc Bonferroni correction). **f** The total antigens of FVIII produced by the patient, WT-KI or FE-KI iPSC-derived ECs in conditioned media and cell lysates was quantified. The data indicate the total number of antigens of FVIII detected per 1 × 10^5^ ECs. The data are presented as the means ± SEMs of three independent experiments. ns indicates nonsignificance (one-way ANOVA with post hoc Tukey’s test).

Genetically modified iPSC lines were differentiated into ECs, which are known as a source of FVIII production^24^. We differentiated ECs from the knock-in iPSC lines and the parental iPSCs via a previously described protocol with minor modifications^19^. On differentiation Day 8, the expression of EC markers was confirmed by qPCR analysis and immunocytochemistry. qPCR analysis revealed that the ECs derived from the knock-in iPSCs and the parental iPSCs expressed similar levels of the EC markers *CD31*, *VWF*, and *VE-cadherin* (Fig. 4a). The immunocytochemical analysis also revealed similar expression of the EC markers CD31 and VWF in ECs derived from the knock-in and parental iPSCs (Fig. 4b).

We examined the mRNA expression levels of *FVIII* in differentiated ECs by qPCR analysis (Fig. 4a). The results revealed no *FVIII* signals in the parental iPSCs or in the ECs differentiated from these cells. In contrast, ECs differentiated from the knock-in iPSCs presented high expression of *FVIII* mRNA, with no significant difference between the WT-KI and FE-KI lines.

We performed FVIII activity assays to investigate the activity of FVIII variants secreted by ECs differentiated from the knock-in iPSC lines. The FVIII activity levels of FE-KI ECs (41.50 ± 3.59% in FE-K1e1, 38.79 ± 4.09% in FE-K1e2) were significantly greater than those of WT-KI ECs (19.17 ± 5.15% in WT-K1e1, 20.88 ± 2.14% in WT-K1e2) (Fig. 4c). We then performed FVIII decay assays to determine the stability of FVIII from each knock-in EC line. We determined the decay rate of FVIII in supernatants obtained from each EC line during cell-free incubation at 37 °C for 24 h (Fig. 4d). The results of the decay assay revealed that the half-life of FVIII secreted by FE-KI ECs was approximately 1.7-fold greater than that of FVIII secreted by WT-KI ECs (Fig. 4e). To determine whether the observed increase in activity was due to increased secretion of FVIII resulting from the introduction of a mutation that enhances FVIII secretion, an ELISA for total FVIII antigen was performed. The results revealed no significant difference in the amount of wild-type FVIII and FE-FVIII in either the medium or the lysate (Fig. 4f).

In summary, these results indicate that iPSCs with the knock-in FE-FVIII successfully differentiated into ECs. Compared with wild-type FVIII, the FE-FVIII secreted by these differentiated ECs exhibited increased activity and stability but not secretion.

### Restoration of FVIII expression in transplanted HA mice

**Fig. 5:**
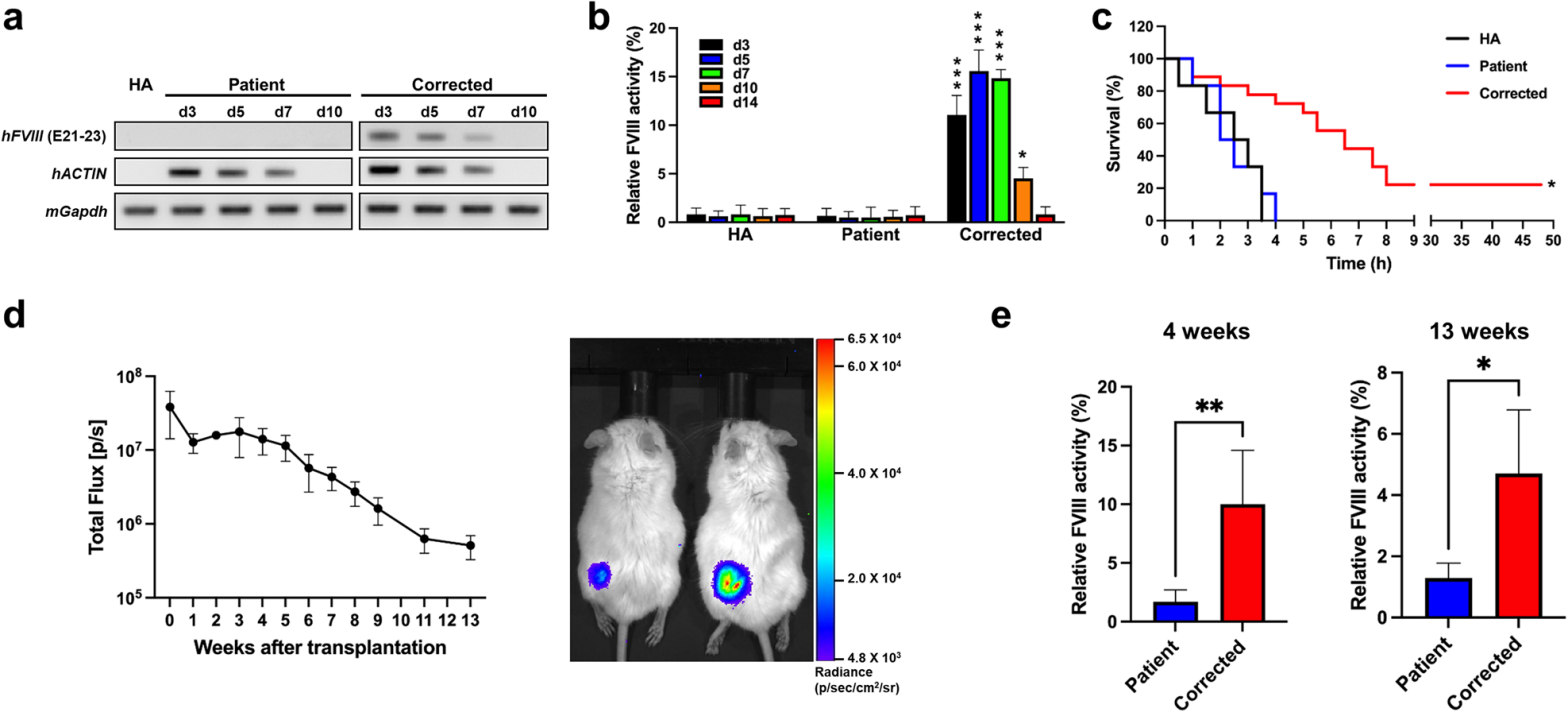
Functional recovery of FVIII in cell-transplanted HA mice. **a** Reverse transcription PCR (RT‒PCR) analysis was used to detect the expression of human *FVIII* in nontransplanted and transplanted HA mice after intravenous transplantation. Human *ACTIN* was used to identify transplanted ECs. Mouse *Gapdh* was used as a control. **b** FVIII activity was measured in blood plasma harvested from nontransplanted and transplanted HA mice after intravenous transplantation. The data represent the activity detected per 1 × 10^6^ ECs. HA (nontransplanted HA mice, *n* = 3 for each day), Patient (*n* = 3 for each day), and Corrected (*n* = 6, 3, 4, 3, and 3). The data are the means ± SEMs. *p < 0.05 and ***p < 0.001 compared with mice with ECs differentiated from parental iPSCs at each timepoint (two-way ANOVA with post hoc Dunnett’s test). **c** Survival curves of nontransplanted and intravenously transplanted HA mice after the tail clip assay. HA (*n* = 6), Patient (*n* = 6), Corrected (*n* = 18). *p < 0.001 compared with the patient group (log-rank test). **d** The survival of subcutaneously transplanted cells in immunodeficient mice was confirmed. ECs differentiated from luciferase reporter KI iPSCs were transplanted subcutaneously into the thighs of immunodeficient mice, and cell survival was monitored by weekly in vivo live imaging up to 13 weeks after transplantation (left panel, *n* = 4). Luciferase activity was still detected 13 weeks after transplantation (right panel). **e** The long-term therapeutic efficacy of subcutaneously transplanted ECs differentiated from parental or corrected iPSCs was determined at 4 and 13 weeks after subcutaneous transplantation into an immunodeficient hemophilia model. Patient (*n* = 3), Corrected (*n* = 4). *p < 0.05, **p < 0.01 compared with the patient group (Student’s *t* test).

To evaluate the in vivo efficacy of FE-FVIII, we transplanted ECs differentiated from parental or FE-K1e1 (corrected) iPSCs into HA mice and harvested blood plasma 3, 5, 7, 10, and 14 days after transplantation. RT‒PCR analysis revealed that *FVIII* was expressed in mice transplanted with corrected ECs but not in those transplanted with ECs differentiated from parental iPSCs (Fig. 5a). FVIII activity assays also revealed no significant difference in FVIII activity between nontransplanted HA mice and mice transplanted with ECs differentiated from parental iPSCs. In contrast, mice transplanted with corrected ECs presented restored FVIII activity until 10 days after transplantation (Fig. 5b).

We subjected the mice to a tail clip challenge 7 days after transplantation. None of the nontransplanted mice or mice transplanted with ECs differentiated from parental iPSCs survived after the tail clip challenge (Fig. 5c), whereas 22.2% of the mice transplanted with corrected ECs survived for at least 2 days, the experimental endpoint.

In previous experiments, we demonstrated the short-term effects of transplanted cells but were unable to verify their long-term effects. This was thought to be due to the insufficient survival of the transplanted cells despite the administration of immunosuppressants, probably due to xenotransplantation (Fig. 5a). Therefore, we developed an immunodeficient hemophilia A mouse, N2G-F8^em1Gmcr,^ to evaluate the long-term effects of the transplanted cells. First, to confirm the long-term survival of transplanted cells in N2G-F8^em1Gmcr^ mice, ECs differentiated from iPSCs constitutively expressing luciferase were transplanted subcutaneously. Luciferase activity was detectable through in vivo live imaging even at 13 weeks posttransplantation (Fig. 5d). Four and thirteen weeks after the transplantation of corrected ECs into N2G-F8^em1Gmcr^ mice, blood samples were collected to measure FVIII activity. The hemophilia phenotype of the corrected EC-treated mice recovered (Fig. 5e).

Taken together, our results indicate that the insertion of *BDD-FVIII* or *FE-FVIII* into the *AAVS1* locus in iPSCs from a patient with HA could produce functional FVIII both in gene-edited iPSCs and in ECs differentiated from these cells. Furthermore, the activity and stability of the FE-FVIII variant were greater than those of WT BDD-FVIII. Immunocompetent HA mice transplanted with corrected ECs recovered FVIII activity for up to 10 days after intravenous transplantation. In contrast, immunodeficient HA mice transplanted with corrected ECs subcutaneously demonstrated long-term FVIII activity recovery for up to 13 weeks after transplantation.

## Discussion

The enhancement of FVIII activity in the blood by transplanted cells is a critical part of the development of hemophilia cell therapies. Our study aimed to address this issue by developing a targeted knock-in strategy to insert a functionally enhanced *FVIII* gene with an EF1α promoter at the human *AAVS1* locus. The knock-in resulted in constitutive expression of the inserted *FVIII* gene in iPSCs derived from a patient with HA and in ECs differentiated from these iPSCs. Moreover, the insertion of a functionally enhanced *FVIII* gene resulted in both increased activity and stability of FVIII in vitro. In addition, HA mice transplanted with corrected ECs presented increased FVIII activity and survival rates after a tail clip challenge, showing ameliorated symptoms of hemophilia compared with those of mice transplanted with ECs derived from iPSCs without *FVIII* knock-in at the *AAVS1* locus.

FVIII is a large plasma glycoprotein composed of a heavy chain (A1–A2–B domains) and a light chain (A3–C1–C2 domains), which together form a heterodimer linked by a divalent metal ion. During the activation of FVIII to its active form, FVIIIa, thrombin cleaves the B domain, separating it from the heavy chain. The B domain of FVIII, the largest and most highly glycosylated domain of FVIII, is not directly required for FVIII activity in the process of blood coagulation. Additionally, FVIII lacking the B domain (BDD-FVIII) is smaller than full-length FVIII (4.3 kb versus 7 kb)^25,26^, making BDD-FVIII a promising therapeutic alternative to the full-length form^27^. However, because of the short half-life (~12 h) and low secretion level of BDD-FVIII^28^, the development of a functionally improved FVIII is a main goal for the treatment of HA. Previous studies focused on improving the secretion or stability of FVIII separately; however, there have been no attempts to improve both parameters simultaneously. We hypothesized that we could produce functionally enhanced FVIII with increased FVIII activity and stability by mutating each domain involved in secretion or stability. We subsequently generated several FVIII variants with mutations that were previously shown to increase either secretion or stability^21,22^. Compared with WT BDD-FVIII, the FVIII variant with the F309S mutation, which is known to increase the secretion of FVIII, exhibited increased activity (Supplementary Fig. 1a). The FVIII variant with the E1984V mutation had an increased half-life (Supplementary Fig. 1b). This result appears to be in line with a previous report^22^, which indicated that substituting the E1984 residue in the A3 domain with the hydrophobic residue valine improved the structural stability of FVIII and reduced the decay rate of FVIIIa. In the case of the FVIII variant with both mutations, both its activity and half-life were found to be improved in comparison with those of wild-type FVIII. Notably, when the amount of FVIII antigen secreted outside the cell was measured, no significant difference was observed between FE-FVIII and WT BDD-FVIII (Fig. 4f). This suggests that, contrary to our expectations, the simultaneous introduction of each mutation results in novel functional attributes rather than the additive effects of each individual mutation. To fully understand these novel properties, comprehensive structural analyses of FE-FVIII will be necessary in further studies.

We obtained iPSCs from a patient with severe HA with an inversion in intron 22 of *FVIII*. A previous study reported that recovery of *FVIII* expression after an intron 22 inversion was corrected in patient-derived iPSCs using CRISPR/Cas9^6^. While the reversal strategy of this *FVIII* inversion successfully restored endogenous *FVIII* expression, it is not applicable to other types of disease-related *FVIII* mutations. In addition, even though correction of the *FVIII* locus restored FVIII expression in ECs derived from gene-edited iPSCs, the low endogenous expression of FVIII in ECs limits the efficacy of cell therapies to cure HA^6^. Among the endothelial cells that express FVIII, sinusoidal endothelial cells in the liver are the primary source^29–31^. The differentiation of PSCs into sinusoidal endothelial cells is a recent development^32,33^ and requires further investigation. To overcome these limitations, we developed a strategy to increase FVIII expression via targeted insertion of the *FVIII* gene with a universal promoter into “safe harbor” sites known to express genes regardless of the cell type. Previous studies have attempted to use engineered nucleases to correct *FVIII* mutations by knocking in the normal *FVIII* gene with a universal promoter at a ribosomal DNA locus, the *FVIII* locus, or the *H11* locus in iPSCs derived from patients with HA^8,9,34^. Another potential safe harbor site for *FVIII* gene insertion is the *AAVS1* locus, which is known to be a fairly stable locus that does not regulate the expression of other nearby genes after transgene insertion, although it is located in the intron of *PPP1R12C*^35^. In addition, the *AAVS1* locus is known to stably express desired genes regardless of the cell type^36^. Because iPSCs are pluripotent and can provide an unlimited source of cells, a strategy to insert a specific gene into a safe harbor site in iPSCs may significantly benefit patients with various types of genetic disorders.

The genetically corrected ECs restored FVIII activity when they were transplanted into HA mice. These results indicate that the functionally enhanced properties of FVIII persisted after genetically corrected iPSC lines differentiated into ECs. However, when transplanted via intravenous injection, the expression of *FVIII* in the transplanted cells in the circulating blood gradually decreased and was undetectable by 10 days after transplantation (Fig. 5a), and the highest level of FVIII activity in the blood was observed 5 days after transplantation, with a subsequent decline in activity (Fig. 5b). In addition, no transplanted cells were identified in the blood of the recipient mice on Day 10 posttransplant.

The observed reduction in *hFVIII* expression in the blood was comparable to that observed for *hACTIN*, which suggests that the decrease in FVIII activity is likely due to the low survival rate of transplanted ECs caused by xenotransplantation. When endothelial colony-forming cells are transplanted subcutaneously, immunodeficient mice exhibit long-term survival of the transplanted cells, whereas immunocompetent mice treated with immunosuppressants exhibit low survival of the transplanted cells despite treatment with immunosuppressants^37^. Our findings are consistent with these results, demonstrating short-term efficacy in immunocompetent mice and long-term efficacy in immunodeficient mice.

Taken together, this knock-in strategy may provide a universal platform to restore FVIII function in patients with various types of *FVIII* mutations via the use of the same sgRNA and donor DNA cassette for ex vivo cell therapy.

## Supplementary information

Supplementary Information
