# Author Correction: Long-term correction of hemophilia A via integration of a functionally enhanced *FVIII* gene into the *AAVS1* locus by nickase in patient-derived iPSCs

**DOI:** 10.1038/s12276-025-01595-x

**Published:** 2025-12-02

**Authors:** Do-Hun Kim, Sang-Hwi Choi, Jin Jea Sung, Sieun Kim, Hanui Yi, Sanghyun Park, Chan Wook Park, Young Woo Oh, Jungil Lee, Dae-Sung Kim, Jong-Hoon Kim, Chul-Yong Park, Dong-Wook Kim

**Affiliations:** 1 Department of Physiology, Yonsei University College of Medicine, 50-1 Yonsei-ro, Seodaemun-gu, Seoul, 03722 Korea; 2 S. Biomedics Co., Ltd, 28 Seongsui-ro 26-gil, Seongdong-gu, Seoul, 04797 Korea; 3 Brain Korea 21 PLUS Program for Medical Science, Yonsei University College of Medicine, 50-1 Yonsei-ro, Seodaemun-gu, Seoul, 03722 Korea; 4 Department of Biotechnology, College of Life Sciences and Biotechnology, Korea University, 145 Anam-ro, Seongbuk-gu, Seoul, 02841 Korea

**Keywords:** Induced pluripotent stem cells, Genetic engineering, Targeted gene repair, Stem-cell research, Haematological diseases

Correction to: *Experimental & Molecular Medicine* 10.1038/s12276-024-01375-z, published online 06 Jan 2025

After online publication of this article, the authors noticed an error in the Figure 2c section.

In Figure 2c, the two lower-left images, which were supposed to represent Nestin immunostaining of FE-K1e1 and FE-K1e2, were inadvertently duplicated. Instead of showing two different experimental results, they represent different regions from the same sample. This error occurred unintentionally during the process of data compilation and figure preparation.

Importantly, this was an unintended mistake and does not affect the scientific results, data interpretation, or overall conclusions of the study.

The authors apologize for any inconvenience caused.

Original Figure 2:
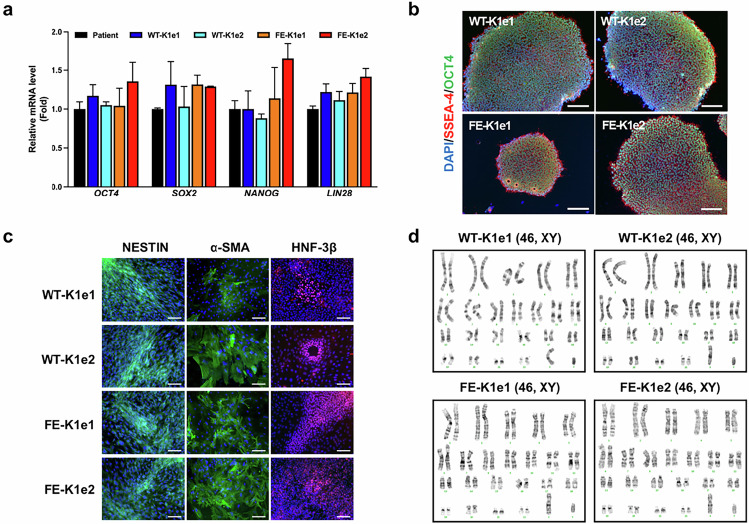


Corrected Figure 2:
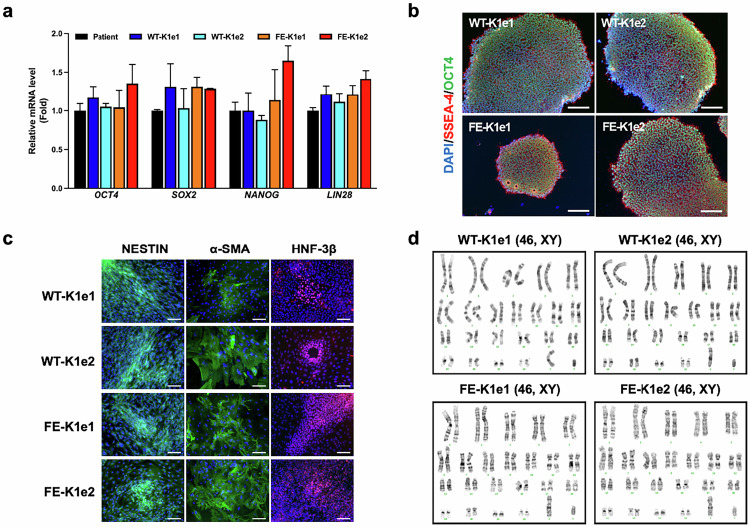


The original article has been corrected.

