# Supplementary material for: Long-term correction of hemophilia A via integration of a functionally enhanced FVIII gene into the AAVS1 locus by nickase in patient-derived iPSCs
Source: Exp Mol Med. 2025 Jan 6;57(1):184–92. doi: 10.1038/s12276-024-01375-z (PMC11799516; doi:10.1038/s12276-024-01375-z)
Supplement: Supplementary file 1 — Supplementary Information [file 12276_2024_1375_MOESM1_ESM.doc]

**Supplementary Information**

**Long-term correction of hemophilia A via integration of a functionally enhanced *FVIII* gene into the *AAVS1* locus by nickase in patient-derived iPSCs**

Do-Hun Kim, Sang-Hwi Choi, Jin Jea Sung, Sieun Kim, Hanui Yi, Sanghyun Park, Chan Wook Park, Young Woo Oh, Jungil Lee, Dae-Sung Kim, Jong-Hoon Kim, Chul-Yong Park, and Dong-Wook Kim

Supplementary Fig. 1. Analysis of FVIII activity level and stability of FVIII variants

Supplementary Fig. 2. Primary PCR screening results of corrected iPSC lines

Supplementary Fig. 3. Off-target analyses in the genetically modified iPSC lines by targeted deep sequencing

Supplementary Table 1. Primer sequences used for genome-based PCR screening and Sanger sequencing

Supplementary Table 2. Sequences of each target site and primers used in off-target amplification

Supplementary Table 3. Antibodies used in this study

Supplemental Figures

**
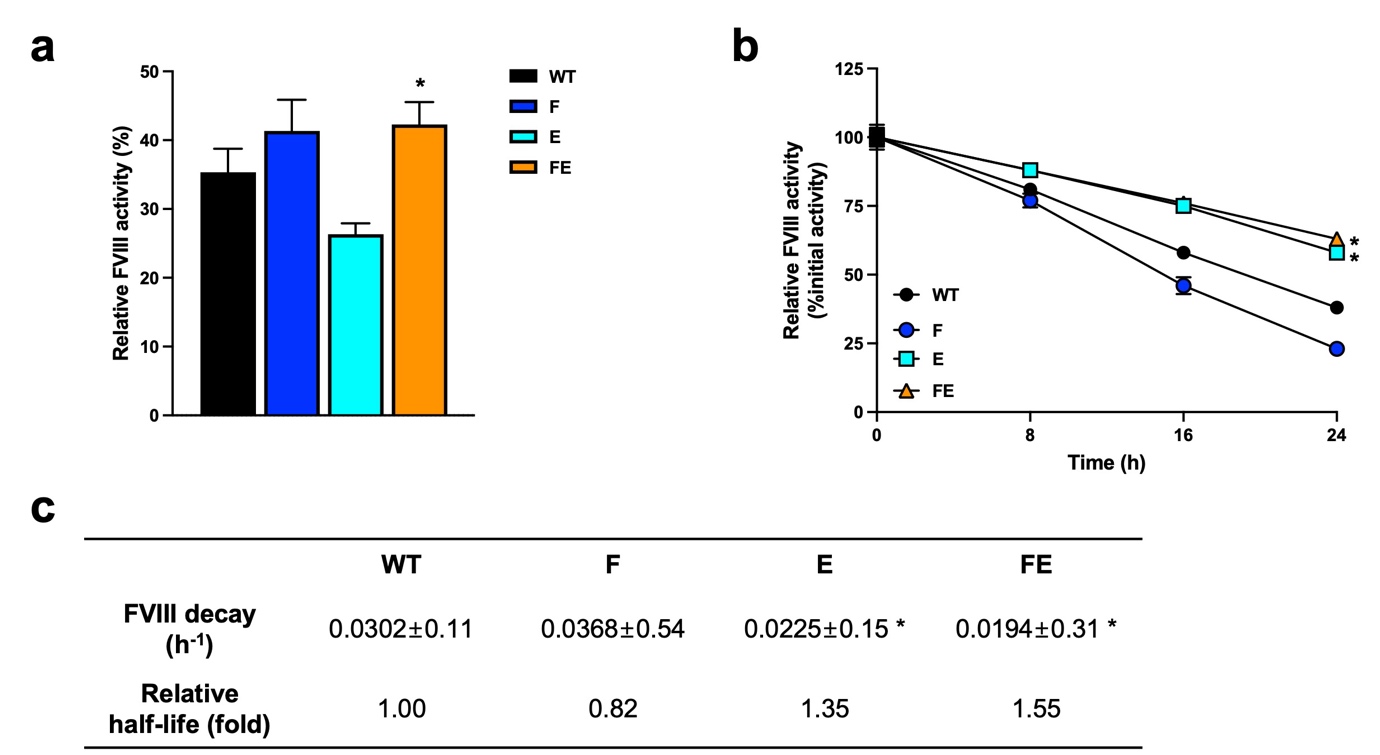
**

**Supplementary Fig. 1. Analysis of FVIII activity level and stability of FVIII variants**

**a** The FVIII activity of FVIII variants. FVIII activity was determined in supernatants obtained from HEK293T cells transfected with plasmids encoding each FVIII variant. WT, wild-type BDD-FVIII; F, F309S-mutated BDD-FVIII; E, E1984V-mutated BDD-FVIII; FE, F309S/E1984V-mutated BDD-FVIII. * *p* < 0.05 compared with BDD-FVIII-transfected cells.

**b** The decay of FVIII variants. FVIII activity in supernatants harvested from transfected HEK293T cells was determined after incubation at 37°C for 0 h, 8 h, 16 h, or 24 h. * *p* < 0.05 compared with WT BDD-FVIII-transfected cells.

**c** Decay rate and relative half-life of FVIII variants. Data are means ± SEM of three independent experiments. SDs for rate decay values are estimated based upon least squares curve fitting and are within approximately 10% of mean values. * *p* < 0.05 compared with WT BDD-FVIII-transfected cells.

**
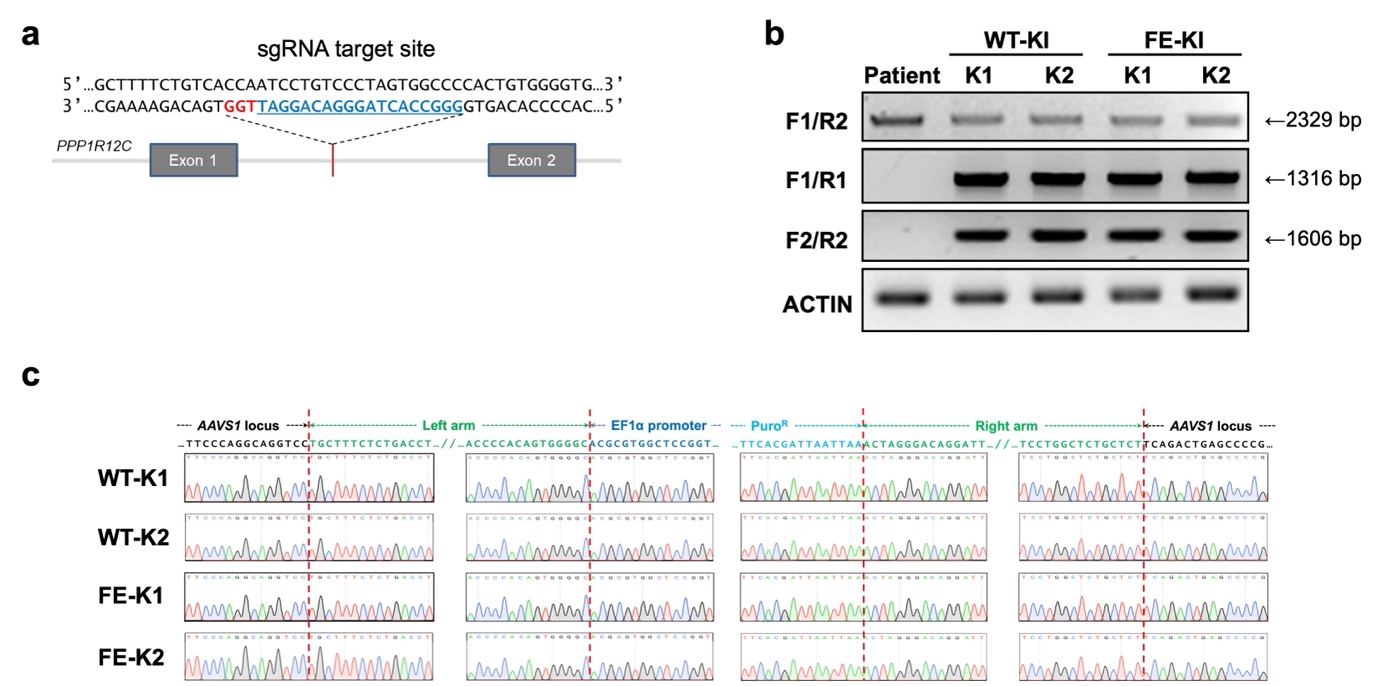
**

**Supplementary Fig. 2. Primary PCR screening results of corrected iPSC lines**

**a** The sgRNA target sequence in the *AAVS1* locus of the *PPP1R12C* gene located on chromosome 19.

**b** PCR-based genotype analysis to confirm the targeted insertion of donor DNA in knock-in iPSC lines. Each primer set represents the 5’ knock-in junction (F1/R1), 3’ knock-in junction (F2/R2), and *AAVS1* locus (F1/R2). ACTIN was used as an internal reference.

**c** Partial sequences of knock-in junctions in the integrated iPSC lines including the template donor DNA. Both arm sequences are shown in green. Donor plasmid sequences started from EF1α promoter and ended from puromycin resistance gene are shown in blue and cyan, respectively. The original genome sequences are shown in black.

**
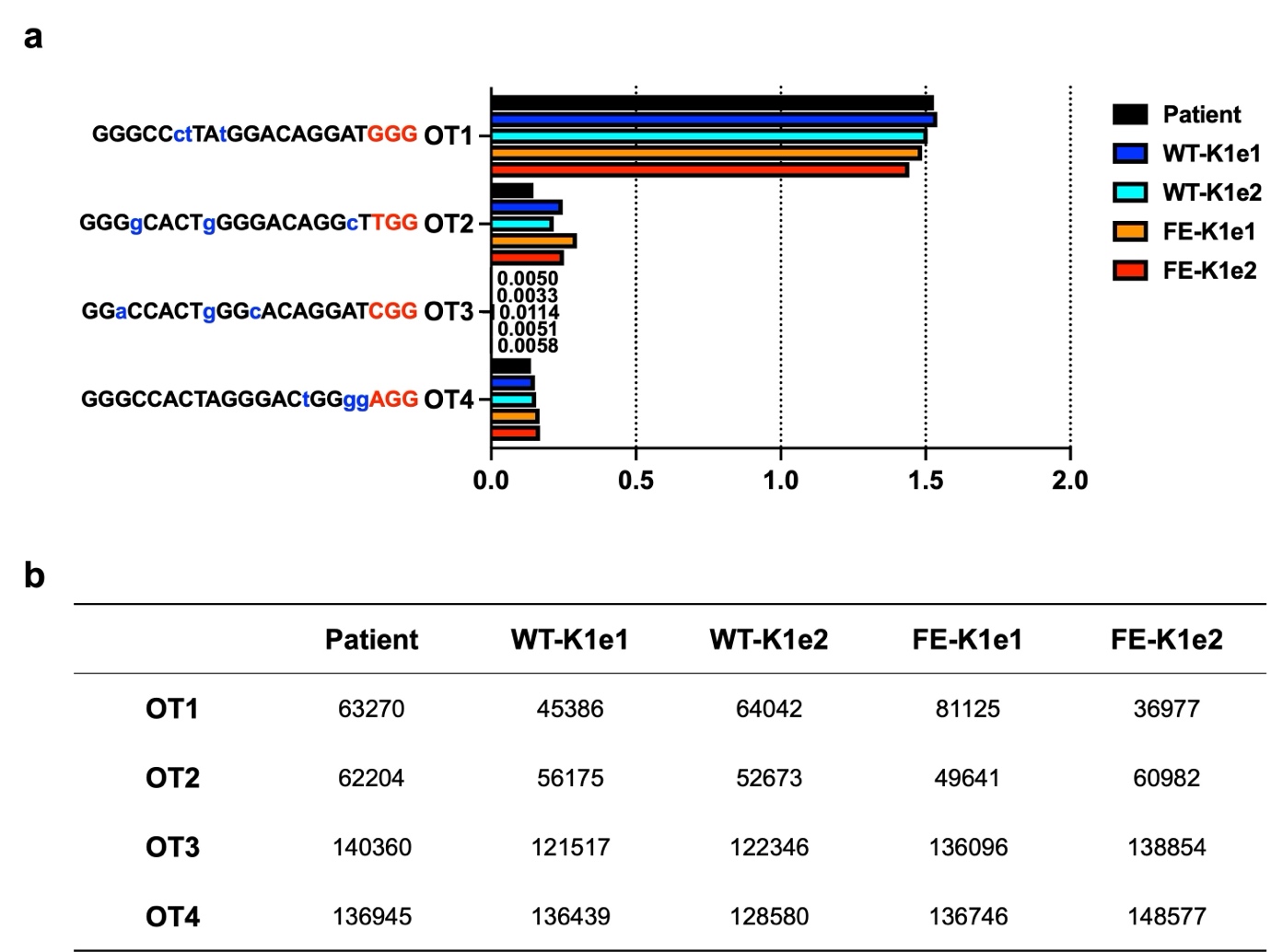
**

**Supplementary Fig. 3. Off-target analyses in the genetically modified iPSC lines by targeted deep sequencing**

**a** Four potential off-target sites differing by up to four nucleotides from the on-target site were examined in the genetically altered clones by targeted deep sequencing. Mismatched nucleotides and PAM sequences (5’-NGG-3’) are shown in blue and red, respectively.

**b** Numbers of total reads.

Supplemental Tables

**Supplemental Table 1. Primer sequences used for genome-based PCR screening and Sanger sequencing**

| **Primers** | **Sequence (5’ to 3’)** | **Used for** |
| --- | --- | --- |
| F1 | TCGACTTCCCCTCTTCCGATG | genotype PCR |
| F2 | GGGGATCAATTCTCTAGAGCTCG | genotype PCR |
| F3 | CCTCATCTCCAGCAGTCAAG | genotype PCR |
| R1 | CCGTTGCGAAAAAGAACGTTCAC | genotype PCR |
| R2 | TGACCAACCATCCCTGTTTT | genotype PCR |
| F309-F | GCCATACATATGTCTGGCAG | genotype PCR |
| F309-R | GATGGCGTTTCAAGACTGGTGG | genotype PCR |
| E1984-F | GGCATGACCGCCTTACTGAAG | genotype PCR |
| E1984-R | CAGCCCAGAACCTCCATCCTC | genotype PCR |
| β-Actin-F | TCACCCACACTGTGCCCATCTACGA | RT-PCR |
| β-Actin-R | CAGCGGAACCGCTCATTGCCAATGG | RT-PCR |
| GAPDH-F | TGCACCACCAACTGCTTAGC | qPCR |
| GAPDH-R | GGCATGGACTGTGGTCATGAG | qPCR |
| OCT4-F | CCTCACTTCACTGCACTGTA | qPCR |
| OCT4-R | CAGGTTTTCTTTCCCTAGCT | qPCR |
| SOX2-F | TTCACATGTCCCAGCACTACCAGA | qPCR |
| SOX2-R | TCACATGTGTGAGAGGGGCAGTGT | qPCR |
| NANOG-F | TGAACCTCAGCTACAAACAG | qPCR |
| NANOG-R | TGGTGGTAGGAAGAGTAAAG | qPCR |
| LIN28-F | AGCCATATGGTAGCCTCATGTCCG | qPCR |
| LIN28-R | TCAATTCTGTGCCTCCGGGAGCAG | qPCR |
| CD31-F | TGCGAATCGATCAGTGGA | qPCR |
| CD31-R | ACCGGGGCTATCACCTTC | qPCR |
| VWF-F | TCGGGCTTCACTTACGTTCT | qPCR |
| VWF-R | CCTTCACTCGGACACACTCA | qPCR |
| VE-cadherin-F | GGTCCCTGAACGCCCTGGTAA | qPCR |
| VE-cadherin-R | GGAGTGGAGTATGGAGTTGGAGCA | qPCR |
| FVIII-exon21-F | CCGGATCAATCAATGCCTGGAG | qPCR and RT-PCR |
| FVIII-exon23-R | ATGAGTTGGGTGCAAACGGATG | qPCR and RT-PCR |
| Mouse Gapdh-F | CATCACTGCCACCCAGAAGACTG | RT-PCR |
| Mouse Gapdh-R | ATGCCAGTGAGCTTCCCGTTCAG | RT-PCR |

**Supplemental Table 2. Sequences of each target site and primers used in off-target amplification**

| **Target site** | **Target Seq. (5’ to 3’)** | **Forward primer Seq.**  **(5’ to 3’)** | **Reverse primer Seq.**  **(5’ to 3’)** |
| --- | --- | --- | --- |
| Off-target 1  (OT1, Chr. 19) | GGGCCCTTATGGACAGGATGGG | GTGCCCGTATCCAGAGTGAT | AGGTGGATGACAAGGTCAGG |
| Off-target 2  (OT2, Chr. 19) | GGGGCACTGGGGACAGGCTTGG | AGGAGGTCAGTCTGGGAGGT | GAGAGGGGCACAAACAGAAG |
| Off-target 3  (OT3, Chr. 15) | GGACCACTGGGCACAGGATCGG | ATGTTGGAAGAGGACGTTGG | TCACATGTCCTCCACCTGTG |
| Off-target 4  (OT4, Chr. 17) | GGGCCACTAGGGACTGGGGAGG | GGGCTATGGGCTTCTCTGA | TGTTTGCTTGCCTCTGACAC |

**Supplemental Table 3**. Antibodies used in this study

| **Antibody** | **Manufacturer** | **Cat #** |
| --- | --- | --- |
| OCT4 | Santa Cruz | SC9081 |
| SSEA-4 | Millipore | MAB4304 |
| NESTIN | Millipore | MAB5326 |
| α-SMA | Sigma | A5228 |
| HNF-3β | Santa Cruz | SC6554 |
| CD31 | BD Bioscience | 555444 |
| vWF | Millipore | AB7356 |
